# Safety of recombinant human platelet-derived growth factor-BB in Augment^®^ Bone Graft

**DOI:** 10.1177/2041731412442668

**Published:** 2012-04-04

**Authors:** Luis A Solchaga, Christopher K Hee, Stephen Roach, Leo B Snel

**Affiliations:** Biomimetic Therapeutics Inc, Franklin, TN, USA

**Keywords:** Platelet-derived growth factor, safety, bone graft substitute, bone repair, arthrodesis rhPDGF-BB.

## Abstract

This article discusses nonclinical and clinical data regarding the safety of recombinant human platelet-derived growth factor-BB as a component of the Augment^®^ Bone Graft (*Augment*). *Augment* is a bone graft substitute intended to be used as an alternative to autologous bone graft in the fusion of hindfoot and ankle joints. Nonclinical studies included assessment of the pharmacokinetic profile of intravenously administered recombinant human platelet-derived growth factor-BB in rat and dog, effects of intravenous administration of recombinant human platelet-derived growth factor-BB in a reproductive and development toxicity study in rats, and chronic toxicity and carcinogenicity of *Augment* in a 12-month implantation model. These studies showed that systemic exposure was brief and clearance was rapid. No signs of toxicity, carcinogenicity, or tumor promotion were observed even with doses far exceeding the maximum clinical dose. Results of clinical trials (605 participants) and commercial use of recombinant human platelet-derived growth factor-BB containing products indicate that these products are not associated with increased incidence of adverse events or cancer. The safety data presented provide evidence that recombinant human platelet-derived growth factor-BB is a safe therapeutic when used in combination products as a single administration during surgical procedures for bone repair and fusion. There is no evidence associating use of recombinant human platelet-derived growth factor-BB in *Augment* with chronic toxicity, carcinogenicity, or tumor promotion.

## Introduction

The platelet-derived growth factor (PDGF) family contains five members found naturally in the body. These are AA homodimer, AB heterodimer, BB homodimer, CC homodimer, and DD homodimer.^1^ PDGF-BB is a homodimer of two antiparallel B-chains covalently linked through two disulfide bonds. While these isoforms have different binding specificities to PDGF cell-surface receptors, PDGF-BB is considered the universal PDGF isoform because it can bind to all known PDGF receptors.^2,3^ PDGF-BB is a primary component of platelets released at sites of injury following platelet activation. PDGF-BB is chemotactic and mitogenic for cells of mesenchymal origin.^2,3^ Additionally, PDGF-BB is proangiogenic, upregulating vascular endothelial growth factor (VEGF) to stimulate new capillary growth.^2^ Through these biological activities, PDGF-BB contributes to tissue repair.

The development of recombinant human PDGF-BB (rhPDGF-BB) for therapeutic purposes focused initially on soft tissue healing with Regranex^®^ (developed by a subsidiary of Johnson & Johnson New Brunswick, NJ, USA; now owned by HealthPoint Biotherapeutics Fort Worth, TX, USA). Later efforts concentrated on regeneration of periodontal bone with GEM 21S^®^ (developed by BioMimetic Therapeutics, Inc., Franklin, TN, USA; now owned by Luitpold Pharmaceuticals, Inc, Shirley, NY, USA). Nonclinical orthopedic studies, including fracture repair in models of impaired healing (geriatric/osteoporotic or diabetic),^4,5^ distraction osteogenesis,^6^ and spine fusion^7^ demonstrated the efficacy of rhPDGF-BB in bone augmentation and the foundation for development of clinical applications.^8–12^

Augment^®^ Bone Graft (*Augment*) comprises an osteoconductive scaffold of β-tricalcium phosphate (β-TCP) and rhPDGF-BB. rhPDGF-BB is a protein of approximately 24.5 kDa produced using recombinant DNA technology in a *Saccharomyces cerevisiae* expression system. The two components are mixed prior to implantation. *Augment* is intended as an alternative to autologous bone graft (autograft) in hindfoot or ankle joint fusion sparing the patient the morbidity of autograft harvest. The average dose of rhPDGF-BB administered in orthopedic clinical trials using *Augment* is 1.8 mg (6 cm^3^ of β-TCP + 6 mL of 0.3 mg/mL rhPDGF-BB); the maximum clinical dose is 2.7 mg (9 cm^3^ of β-TCP + 9 mL of 0.3 mg/mL rhPDGF-BB).

Due to its biological activity, altered expression of PDGF-BB has been associated with concerns of potential tumor promotion.^13,14^ However, as discussed below, the emerging data appear to provide reassurance about the safety of rhPDGF-BB. This is, in part, due to the fundamental differences between the local single administration of rhPDGF-BB in a combination device such as GEM 21S or *Agument* and the continuous deregulated expression of PDGF-BB in certain cancers.

Here, we present the latest nonclinical studies and review the data on the safety of rhPDGF-BB.

## Materials and methods

Testing was conducted in accordance with guidance from the International Organization for Standardization (ISO), International Conference on Harmonisation (ICH), and United States Pharmacopeia (USP). Animal studies were performed at facilities accredited by the Association for Assessment and Accreditation of Laboratory Animal Care (AAALAC) under protocols approved by the Institutional Animal Care and Use Committee (IACUC) review (Table 1). rhPDGF-BB was provided by BioMimetic Therapeutics, Inc. β-TCP granules were obtained from Cam Bioceramics (Leiden, The Netherlands).

**Table 1.** Nonclinical safety studies

	Study	Results
Pharmacokinetics Rat	Pharmacokinetics of rhPDGF-BB in Sprague Dawley rats following intravenous administration	C_max_: 6161.2 ng/mL
10 × maximum clinical dose		T_max_: 1.0 min
		CL: 17.5 mL/min/kg
Pharmacokinetics Dog	Pharmacokinetics study in male beagle dogs following single intravenous administration of rhPDGF-BB	C_max_: 14,603.8 ng/mL
23 × maximum clinical dose		T_max_: 1.0 min
		CL: 423.3 mL/min/kg
Reproductive and developmental toxicity	rhPDGF-BB: an intravenous injection teratology study in the rats	No maternal toxicity
210 × maximum clinical dose		No fetal toxicity
Carcinogenicity and chronic toxicity	Evaluation of the chronic toxicity and carcinogenicity of rhPDGF-BB mixed with β-TCP (Augment^®^ Bone Graft) implanted in a rat model	No carcinogenicity
4 × maximum clinical dose		No toxicity
		Transient local reaction to implanted material observed in both groups

rhPDGF-BB: recombinant human platelet-derived growth factor-BB; β-TCP: β-tricalcium phosphate; C_max_: maximum concentration observed; T_max_: time point at which the maximum concentration was observed; CL: clearance.

### Pharmacokinetics

Two studies were carried out to evaluate circulating levels of rhPDGF-BB administered as a single intravenous (IV) dose. In the first study, 48 Sprague Dawley rats were distributed into two groups that received a single IV dose of either 0.44 mg/kg rhPDGF-BB or 20 mM sodium acetate. Serum samples were collected at baseline, 1, 5, 10, and 20 min and 1, 4, 8, 24, 48, 72, 96, and 168 h post dose (n = 6 per group per time point). In the second study, eight beagle dogs were distributed into two groups that received a single IV bolus dose of either 1.0 mg/kg of rhPDGF-BB or 20 mM sodium acetate. Samples were collected at the same time points listed above (n = 4 per group per time point). For both studies, the concentration of rhPDGF-BB was determined by enzyme-linked immunosorbent assay (ELISA). Pharmacokinetic analyses were performed using a noncompartmental module of WinNonlin^®^ (Pharsight, Cary, NC, USA).

### Reproductive and developmental toxicity

The potential toxicity of rhPDGF-BB was studied by daily IV administration in gravid rats over 21 days of gestation. Three groups of 22 female rats received single daily doses of vehicle control (20 mM sodium acetate, pH 6.0) or rhPDGF-BB at a dose of either 40 or 400 µg/kg/day via IV injection for 21 days. On day 21 of gestation, all rats were euthanized and a gross pathological examination was performed. The reproductive tract of each dam was dissected, the ovaries were removed, and the corpora lutea were counted. The gravid uterus was weighed, the uterine contents were examined, and the number and position of live and dead fetuses were recorded. Each fetus was weighed and given a detailed external examination. Additional groups of six rats per group were included for toxicokinetic analysis at baseline and day 21. Serum rhPDGF-BB concentration and antibody formation against rhPDGF-BB were determined using ELISA.

### Carcinogenicity and chronic toxicity

In all, 300 (150 male, 150 female) Sprague Dawley rats were distributed into three groups. β-TCP was combined with either 0.3 mg/mL of rhPDGF-BB (test) or sodium acetate (control). Aliquots of 200 µL of test or control article were implanted adjacent to the femur, underneath the muscle; the third group received sham surgery. Animals were euthanized after 30 (n = 10 per group per sex), 180 (n = 10 per group per sex), or 365 days (n = 30 per group per sex). Macroscopic and microscopic evaluations of toxicity and tumor incidence were performed. Serum was collected for hematology and clinical chemistry determinations. Anti–PDGF-BB antibody formation was evaluated by ELISA.

## Results

### Pharmacokinetics

In the rat study, with an average delivered dose of 440.5 µg/kg, the concentration of rhPDGF-BB in serum decreased between 5 min and 1 h. After 1 h, the serum levels of rhPDGF-BB were below the level of quantitation (<0.156 ng/mL). The T_max_ was observed at 1 min with a C_max_ of 6161.2 ng/mL. The Area under the curve from time 0 to 4 hours AUC_0–4_ was 380.7 h × ng/mL, and the clearance was 17.5 mL/min/kg (Table 1). Following administration of the 1.06 mg/kg IV dose in the canine model, C_max_ (14,603.8 ng/mL) occurred 1 min post dose. Half-life was 0.92 h. Inferred Area under the curve AUC_inf_ was 2504.4 h × ng/mL, and the clearance was 423.3 mL/min/kg (Table 1).

### Reproductive and developmental toxicity

No treatment-related mortality or significant adverse effects were observed. Effects on body weight, body weight gains, and food consumption were unremarkable. The gross pathological assessment was unremarkable in all treatment groups. The uterine parameters assessed (i.e. pregnancy rate, number of corpora lutea, implantation sites, live and dead fetuses, sex ratio, resorptions, and pre- and postimplantation losses) were unaffected in all treatment groups. Fetal weights were unaffected by treatment. The incidence of litters and fetuses with major malformations was unaffected by the treatment across all treatment groups. The incidence of minor external and visceral anomalies was unaffected by rhPDGF-BB. Rat plasma samples were assayed for rhPDGF-BB using an ELISA. The plasma levels of rhPDGF-BB in all dams and fetuses were below the level of detection (<0.625 mg/mL). No anti–rhPDGF-BB antibodies were detected in the 45 samples tested except in one pretreatment sample of one of the dams.

### Carcinogenicity and chronic toxicity

No treatment-related mortality or effects on the clinical condition of the rats were observed. No remarkable test article–related changes in the body weight or body weight gain were observed. No significant changes in urinalysis parameters across treatment groups and gender were observed. Similarly, no significant changes in bone marrow parameters across treatment groups and gender were observed. There were no test article–related microscopic findings on days 30, 180, or 365 of the study. On day 30, minimal foreign body granulomas containing material consistent with surgical sutures were present at the implant site across groups. Minimal to mild granulation tissue was noted at the implant site in animals from the control and the test article groups on day 30, day 180, and day 365. Because this was observed in both groups, it was likely a local reaction to the β-TCP. There were no test article–related neoplastic microscopic observations noted in either sex on day 365. None of the animals treated with the test article were positive for anti–PDGF-BB antibodies.

## Discussion

### Nonclinical studies

The safety of rhPDGF-BB alone or in combination with β-TCP has been demonstrated in a comprehensive battery of in vivo and in vitro studies. The test materials were not mutagenic, hemolytic, cytotoxic, pyrogenic, or allergenic, and there was no evidence of either local or systemic toxicity.^15,16^ Repeated daily administration of rhPDGF-BB by IV injection throughout the gestation period did not have any adverse effect in the dams or their progeny. A 1-year toxicity study demonstrated that implantation of rhPDGF-BB in combination with β-TCP did not elicit any adverse effects, tumor formation, or untoward immune reaction against the device. These outcomes highlight the safety of *Augment*, which is an implantable combination product intended for a single local administration for orthopedic tissue repair.

### Regulation of local and systemic availability and clearance

PDGF is released from platelets at sites of injury and has a localized stimulatory effect on the wound healing process. Intrinsic clearance mechanisms limit systemic availability and regulate the local activity of PDGF. The presence of a plasma PDGF–binding protein was described in 1984.^17–19^ Characterization of the interaction of PDGF with plasma proteins determined that this plasma protein was α_2_-macroglobulin.^18,19^ The α_2_-macroglobulin is present in two forms: a native and a transformed conformation, which occurs after protease activation. The receptor-mediated effects of PDGF (chemotaxis and mitogenesis) are inhibited by binding to α_2_-macroglobulin.^17,18^ Interaction with α_2_-macroglobulin also regulates the clearance of PDGF. PDGF binds preferentially to the conformationally transformed α_2_-macroglobulin,^20^ leading to rapid clearance of the complex through the low-density-lipoprotein-receptor-related protein (LRP) pathway.^20,21^ Bonner et al.^22^ proposed that native α_2_-macroglobulin binds PDGF, serving as a reservoir of PDGF to be released in the healing environment (i.e. sites of injury with low pH) but keeps it sequestered from systemic exposure. The inflammatory response and presence of proteases in sites of injury increase the conformationally transformed α_2_-macroglobulin leading to preferential binding of PDGF and rapid clearance from the circulation.

The results of the in vivo studies using radio-labeled rhPDGF-BB (^125^I-rhPDGF-BB) demonstrated that 60% to 70% of the locally administered rhPDGF-BB was released from the implantation site in the first 60 min, followed by a slower, sustained release over several days.^16^ An additional study to assess the pharmacokinetics differences between IV administration of ^125^I-rhPDGF-BB and intramuscular (IM) implantation of ^125^I-rhPDGF-BB combined with β-TCP determined that the systemic bioavailability was similar by both routes of administration.^16^ Two studies with nonlabeled rhPDGF-BB delivered via IV administration to rats and dogs corroborate the findings of the studies performed with isotope-labeled rhPDGF-BB. In these studies, determination of rhPDGF-BB levels in serum was performed by ELISA. The half-life, T_max_, C_max_, and clearance were comparable to those reported in previous studies.^16^

### Tumorigenic potential of PDGF-BB

PDGF-B shares sequence homology with the simian sarcoma virus oncogene, *v-sis*, and its cellular counterpart, *c-sis.*^23,24^ Cellular transformation induced by viral infection by *v-sis* is through the constitutive expression of the protein.^25^ Nonclinical evaluations, in vitro and in vivo, have demonstrated that chronic, constitutive expression of *v-sis* and PDGF-BB results in cellular transformation.^25,26^ Although these observations, combined with the identification of high levels of PDGF-BB and PDGF receptors in certain tumors,^13,14^ associate PDGF-BB with a potential cancer risk, there are critical differences between viral infection by *v-sis* and the single-time administration of rhPDGF-BB in *Augment*. Removal of *v-sis* or PDGF-BB stimulus (i.e. gene silencing, neutralizing antibodies, chemical inhibition) results in reversal of the transformation.^27–30^ This suggests that these tumors are PDGF-BB dependent, requiring continuous exposure to PDGF-BB to maintain their transformed state. Furthermore, these observations may reflect autocrine or paracrine stimulation of proliferation rather than deregulation of the cell cycle that is the hallmark of transformed cells.^31^

### Clinical experience with rhPDGF-BB

Although some apprehension has been voiced about the risk of cancer formation or promotion with the use of growth factors, data from clinical investigations do not support such concerns. For example, the final analysis of a large study on patients treated with a topical gel containing rhPDGF-BB (Regranex) found no increased risk of cancer incidence or cancer mortality with administration of rhPDGF-BB.^32^ This is reinforced by the data from a multicenter, randomized, controlled clinical trial comparing *Augment* and autograft (2:1 randomization scheme) with 414 study subjects and a 12-month follow-up. Five cancer events were reported in this clinical trial: three in the *Augment* group and two in the autograft group (1.1% and 1.4% incidence, respectively); none of the tumors in the *Augment* group were at or near the site of implantation (Table 2). The cancer events from six additional clinical studies using rhPDGF-BB and β-TCP are listed in Table 2. The cancer incidence in rhPDGF-BB–treated subjects was 0.7% compared to 1.3% in control subjects. None of the 605 patients treated with rhPDGF-BB in these seven clinical trials had any serious device-related adverse events, immunologic sequelae, or other negative reactions attributable to the product.

**Table 2.** Reported adverse events and cancers in clinical trials using rhPDGF-BB/β-TCP combination products

Indication	No. of subjects (rhPDGF-BB + β-TCP/control)	Country	Patients with device-related adverse events	Reported cancers	Comments
Periodontal defects^8^	180 (121/59)	USA	18 (10.0%)	1	rhPDGF-BB + β-TCP: 0 (0.0%)
			[13 (10.7%)/5 (8.5%]		β-TCP alone: 1 (1.7%)
					1. Basal cell carcinoma
Distal radius fracture	40 (20/20)	Sweden	0 (0%)	0	—
			0 (0%)/0 (0%)		
Foot and ankle arthrodesis^10^	60 (60/0)	Canada	4 (6.7%)	1	*Augment*: 1 (1.7%)
			[4 (6.7%)/— (—)]		1. Colon cancer
Foot and ankle arthrodesis	10 (10/0)	Canada	0 (0%)	0	—
			[0 (0%)/— (—)]		
Hindfoot and ankle arthrodesis^12^	20 (14/6)	USA	0 (0%)	0	—
			[0 (0%)/0 (0%)]		
Hindfoot and ankle arthrodesis (submitted to *Journal of Bone and Joint Surgery*)	414 (272/142)	USA	12 (2.9%)	5	*Augment*: 3 (1.1%) Prostate cancer Prostate cancer Infiltrating lobular carcinoma
		Canada	6 (2.2%)/6 (4.2%)		
					Autograft: 2 (1.4%) Renal carcinoma Endometrial cancer
Foot and ankle arthrodesis	108 (108/0)	Netherlands	6 (5.6%)	0	—
		Belgium	[6 (5.6%)/— (—)]		
		Austria			
		France			
Total	832 (605/227)		40 (4.8%)	7	rhPDGF-BB + β-TCP: 4 (0.7%)
			[29 (4.8%)/11 (4.8%)]		Control: 3 (1.3%)

rhPDGF-BB: recombinant human platelet-derived growth factor-BB; β-TCP: β-tricalcium phosphate

GEM 21S, which was approved by the Food and Drug Administration (FDA) periodontal bone regeneration, is essentially identical to *Augment*. Upon review of all the available information from more than 150,000 implanted units, only one potential cancer-related event has been reported. The only commercial use of *Augment* has been in Canada where the product was approved in 2009 for foot and ankle fusions. Between 2009 and 2011 there were over 200 kits of *Augment* sold without receipt of a single report of adverse events (including cancer).

## Conclusion

The use of rhPDGF-BB and *Augment* has been evaluated for potential toxicity in a variety of studies without suggestion of toxicity, carcinogenicity, or mutagenicity after a single administration as an implantable device/drug combination product. The biological activity of rhPDGF-BB is regulated by α_2_-macroglobulin resulting in brief systemic exposure and rapid elimination of the protein by normal metabolic routes. Data from multiple clinical trials with *Augment* and GEM 21S and use of GEM 21S in clinical practice indicate that cancer incidence is not increased with clinical application of rhPDGF-BB.
